# *Yersinia enterocolitica* Outbreak Associated with Ready-to-Eat Salad Mix, Norway, 2011

**DOI:** 10.3201/eid1809.120087

**Published:** 2012-09

**Authors:** Emily MacDonald, Berit Tafjord Heier, Karin Nygård, Torunn Stalheim, Kofitsyo S. Cudjoe, Taran Skjerdal, Astrid Louise Wester, Bjørn-Arne Lindstedt, Trine-Lise Stavnes, Line Vold

**Affiliations:** Norwegian Institute of Public Health, Oslo, Norway (E. MacDonald, B.T. Heier, K. Nygård, A.L. Wester, B.-A. Lindstedt, T.-L. Stavnes, L. Vold);; Norwegian Food Safety Authority, Oslo (T. Stalheim);; and Norwegian Veterinary Institute, Oslo (K.S. Cudjoe, T. Skjerdal)

**Keywords:** Disease outbreaks, Yersinia enterocolitica, vegetables, salad, foodborne, bacteria, enteric infections, Norway, bacteria, enteritis

## Abstract

In 2011, an outbreak of illness caused by *Yersinia enterocolitica* O:9 in Norway was linked to ready-to-eat salad mix, an unusual vehicle for this pathogen. The outbreak illustrates the need to characterize isolates of this organism, and reinforces the need for international traceback mechanisms for fresh produce.

Yersiniosis, a notifiable disease in Norway, is the fourth most common cause of acute bacterial enteritis registered by the Norwegian Surveillance System for Communicable Diseases. Approximately 30 domestic cases are reported annually (2010 incidence rate 0.5 cases/100,000 population). In Norway, >98% of cases of *Yersinia enterocolitica* infection are caused by serotype O:3, which is also the dominant serotype in Europe, Japan, and parts of North America (*1*). Infection by *Y. enterocolitica* is often associated with ingestion of pork because pigs commonly harbor the pathogenic serotypes O:3 and O:9 (*1*). Recent foodborne outbreaks have been associated with pork products (*2*,*3*) and pasteurized milk (*4*).

In Norway, fecal specimens from patients who have acute gastroenteritis are routinely tested for the presence of *Y. enterocolitica*. Presumptive *Y. enterocolitica* O:3 and O:9 isolates are sent by primary laboratories to the National Reference Laboratory (NRL) at the Norwegian Institute of Public Health, where they are routinely verified, serotyped against a range of O antisera, biotyped if relevant, and tested for *Yersinia* virulence plasmid (pYV). If the strains are pathogenic *Y. enterocolitica*, they are typed by use of multiple-locus variable-number tandem repeat analysis (MLVA), using multicolor capillary electrophoresis (*5*). In March 2011, a multidisciplinary investigation was initiated after the NRL received 5 isolates of *Y. enterocolitica* O:9 from humans in disparate areas of the country. All had an identical MLVA profile, which had not been previously seen in Norway. An international request for information produced no reports of similar outbreaks.

## The Study

**Figure 1:**
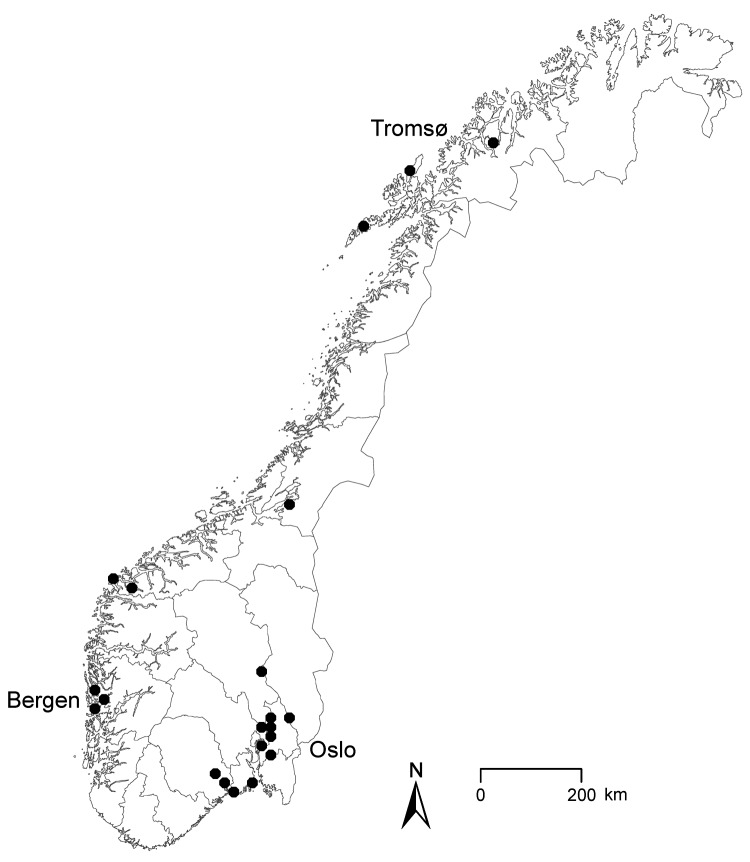
Geographic distribution of 21 outbreak cases (dots) of *Yersinia enterocolitica* O:9 infection, Norway, February–April 2011. Scale bar represents 100 kilometers.

**Figure 2:**
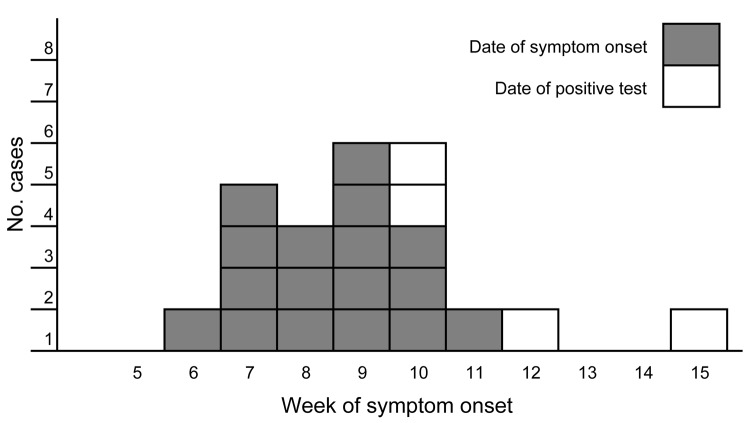
Week of symptom onset or positive test result for 21 persons with *Yersinia enterocolitica* O:9 infection, Norway, 2011. Dark gray, date of symptom onset for 17 case-patients; light gray, date of positive test result for 4 case-patients for whom the date of symptom onset was not available.

A confirmed case-patient was defined as a person in Norway after January 1, 2011, who had laboratory-confirmed *Y. enterocolitica* O:9 infection that matched the MLVA profile of the outbreak strain. By May 5, the NRL had registered 21 outbreak case-patients (median age 37 years [range 10–63 years]), of whom 15 were female. Case-patients resided in 10 geographically dispersed municipalities throughout the country (Figure 1). Most case-patients became ill during February 7–March 20 (Figure 2).

We interviewed the first 7 case-patients by using a standard trawling questionnaire, which was subsequently shortened and used to test hypotheses in a matched case–control study. Two of the case-patients initially interviewed stated that they had eaten a specific blend and brand of salad mix. Nine case-patients were included in the case–control study. Three controls for each case-patient were selected from the National Population Register. Controls and case-patients were matched by age, sex, and municipality of residence and were interviewed in person or by telephone.

In the univariate analysis, ready-to-eat salad mix, diced ham, chicken breast, and arugula were statistically associated with illness (Table). Diced ham and chicken breast were rejected as likely sources of infection because the case-patients reported eating different brands purchased at different stores. Because arugula was an ingredient in the salad mix in question, it was not possible to otherwise distinguish it in the univariate analysis from the salad mix itself. The only food item that remained significant in the final multivariable model was salad mix. Sixteen of the identified case-patients reported having eaten salad mix.

**Table:** Results from univariate conditional logistic regression analyses of a *Yersinia enterocolitica* outbreak, Norway, February–April 2011

Exposure	No. cases, n = 9	No. controls, n = 26*	Matched odds ratio† (95% CI)	p value
Ready-to-eat salad mix	6	3	13.7 (1.6–116.3)	0.02
Diced ham, ham pieces	5	3	6.3 (1.2–32.9)	0.03
Chicken breast	8	9	10.0 (1.2–83.6)	0.03
Arugula	7	8	9.8 (1.2–83.6)	0.04
Pork chops	4	3	8.4 (0.9–78.6)	0.06
Bean sprouts	3	1	8.2 (0.8–79.3)	0.07
Sugar peas	6	8	3.7 (0.9–16.1)	0.08
Iceberg lettuce	8	13	6.6 (0.8–57.5)	0.09

*n = 25 for ready-to-eat salad mix.

We traced the suspected salad mix to a single Norwegian company. Under the auspices of the Norwegian Food Safety Authority, we conducted an environmental investigation, including a traceback investigation and a review of production and cleaning procedures at the company. The suspected salad mix contained 4 salad green types: arugula, radicchio rosso, iceberg lettuce, and endive. These ingredients came, unprocessed, from 12 suppliers in 2 European countries. After delivery to the company in Norway, the greens were washed in 2 cold water baths, cut, and packaged. We found no indications of inadequate routines for ingredient control, hygiene, or sampling within Norway. We identified radicchio rosso, a leaf chicory, as the likely source of infection because it can be stored for several months and was the only ingredient included in the suspected salad mix that had delivery, production, and storage dates consistent with the outbreak period. The company in Norway traced the radicchio rosso to 1 of 3 possible growers in 1 European country but was not able to identify the source of contamination. The Norwegian company voluntarily withdrew all salad mixes containing radicchio rosso from the market. After withdrawal of the implicated ingredients, no new outbreak cases were reported.

We collected 37 food samples from the homes of case-patients, retail locations, and the company that processed the salad mix products and analyzed them at the Norwegian Veterinary Institute. We cold-enriched all samples for 21 days and analyzed them according to conventional culture methods. Using PCR, we examined all enriched broths and isolated colonies for the *ail* gene, an indicator of pathogenic *Y. enterocolitica* that was found in the outbreak strain (*6*,*7*) (www.nmkl.org/kronologisk.htm). We cultured *Y. enterocolitica*–positive samples to isolate *Yersinia* colonies. We isolated presumptive *Yersinia* spp. from 11 of the salad ingredients, including 2 strains that were consistently positive for the *ail* gene and that were isolated from mixed salad and radicchio rosso samples obtained directly from the company. All of the specimens were sent to the NRL for characterization to the species level, biotyping, serotyping, and testing for pYV. The isolate found in the mixed salad sample was *Y. enterocolitica* biotype 1A. It did not agglutinate in antiserum 0 through 34 and was pYV negative, whereas the radicchio rosso isolate was identified as *Y. kristensenii* and agglutinated in O:11,24 antiserum.

## Conclusions

This outbreak of *Y. enterocolitica* was associated with ingestion of ready-to-eat salad mix. Despite a thorough traceback investigation, the likely source of contamination for the outbreak, radicchio rosso, remains unproved. As found in many outbreaks linked to salad products (*8*–*10*), more women were affected than men or children. The outbreak was detected rapidly through systematic characterization of *Y. enterocolitica* isolated from humans to a level that enabled identification of an outbreak of non-O:3 *Y. enterocolitica* infections, which were linked by MLVA. Few European countries regularly type *Y. enterocolitica*, which might explain why international requests for information produced no similar reports from other countries.

To our knowledge, isolates of pathogenic *Y. enterocolitica* are seldom recovered from food samples. Previous survey studies of *Yersinia* spp. in prepared salads have recovered primarily nonpathogenic and environmental strains, such as *Y. enterocolitica* biotype 1A and *Y. kristensenii* (*11*,*12*). In addition, evidence is increasing that the *ail* gene is not always associated with pathogenic *Y. enterocolitica*, suggesting that *ail*-based detection methods for potentially pathogenic *Y. enterocolitica* in food should be supplemented by isolating the strain itself for further characterization or by investigating for other virulence markers. Strains of *Y. enterocolitica* biotype 1A, which are considered nonpathogenic, harbor the gene (*13*,*14*), whereas it might be absent from some pathogenic strains (*15*). Although we could not conclusively link any of the isolates from the salad ingredients to the human *Y. enterocolitica* isolates, finding nonpathogenic *Yersinia* in packaged salads reinforces that the environment of this food product is conducive to the persistence of the bacterium. No further outbreak cases were reported after the salad withdrawal, supporting our conclusion.

The necessity of epidemiologic, environmental, and traceback investigations in addition to microbiological investigation in outbreak situations cannot be underestimated. Considering the increasing number of outbreaks associated with fresh produce, improving traceback among these products should be emphasized. Despite the ultimately inconclusive traceback investigation, this outbreak shows the value of targeted surveillance and microbiological typing as a means for quickly identifying and investigating small foodborne disease outbreaks with international implications.
